# Supplemental Oxygen Protects Heart Against Acute Myocardial Infarction

**DOI:** 10.3389/fcvm.2018.00114

**Published:** 2018-08-28

**Authors:** Anjali M. Prabhat, M. Lakshmi Kuppusamy, Shan K. Naidu, Sarath Meduru, Praneeth T. Reddy, Abishai Dominic, Mahmood Khan, Brian K. Rivera, Periannan Kuppusamy

**Affiliations:** ^1^Departments of Radiology and Medicine, Geisel School of Medicine, Dartmouth College, Hanover, NH, United States; ^2^Dorothy M. Davis Heart and Lung Research Institute, Columbus, OH, United States; ^3^Department of Emergency Medicine, Ohio State University Wexner Medical Center, Columbus, OH, United States

**Keywords:** supplemental oxygen, oxygen cycling, myocardial infarction, ischemia-reperfusion injury, oximetry

## Abstract

Myocardial infarction (MI), which occurs often due to acute ischemia followed by reflow, is associated with irreversible loss (death) of cardiomyocytes. If left untreated, MI will lead to progressive loss of viable cardiomyocytes, deterioration of cardiac function, and congestive heart failure. While supplemental oxygen therapy has long been in practice to treat acute MI, there has not been a clear scientific basis for the observed beneficial effects. Further, there is no rationale for the amount or duration of administration of supplemental oxygenation for effective therapy. The goal of the present study was to determine an optimum oxygenation protocol that can be clinically applicable for treating acute MI. Using EPR oximetry, we studied the effect of exposure to supplemental oxygen cycling (OxCy) administered by inhalation of 21–100% oxygen for brief periods (15–90 min), daily for 5 days, using a rat model of acute MI. Myocardial oxygen tension (pO_2_), cardiac function and pro-survival/apoptotic signaling molecules were used as markers of treatment outcome. OxCy resulted in a significant reduction of infarct size and improvement of cardiac function. An optimal condition of 30-min OxCy with 95% oxygen + 5% CO_2_ under normobaric conditions was found to be effective for cardioprotection.

## Introduction

A myocardial infarction (MI) or a “heart attack,” in which there is an obstruction of blood flow to the coronary arteries, is commonly followed by ischemia-reperfusion (I-R) injury upon restoration of circulation. This “one-two punch” often results in widespread cardiac tissue damage and irreversible loss of cardiomyocytes (heart muscle cells), which can lead to cardiac dysfunction and eventual heart failure. Therapeutic approaches that limit the long-term tissue damage, loss of viable cardiomyocytes, and remodeling of the heart would be beneficial toward improving clinical outcomes post-MI.

It is reasonable to assume that administration of pure oxygen during and after a cardiac event to increase blood oxygen delivery to the affected heart tissue would reduce infarct size and salvage at-risk myocardial tissue. This, in turn, is expected to lead to improvement in functional recovery. Under most circumstances, provision of supplemental oxygen to suspected MI patients by emergency responders is routine (1). Until recently, some guidelines recommended regular administration of oxygen for the treatment of MI (2, 3). This position has changed, however, so that continued treatment with oxygen is only recommended under certain circumstances and not for cases of uncomplicated MI (1, 4). It is likely that this change in treatment protocol is due to known hemodynamic side effects associated with hyperoxygenation. Hyperoxygenation of patients with acute MI results in a rise in arterial blood pressure and a reduction in cardiac output (5, 6). These changes have been attributed to decreases in heart rate and stroke volume (7), and an increase in vascular resistance (8–10). Hyperoxygenation is also a powerful stimulus of coronary circulation and vasoconstriction (11).

In addition to normobaric oxygen, hyperbaric oxygen (HBO) has also been investigated as a potential therapeutic measure for MI. A study by Cameron et al. reported that the hemodynamic effects of oxygen therapy in MI patients at normobaric pressure (1 ATA, atmospheres absolute) were enhanced upon an increase to 2 ATA (8). In 1998, the “HOT MI” study attributed greater left-ventricular ejection fraction in the HBO-treated group to increased myocardial salvage when compared to the non-treated subjects (12). In 1969, Ashfield and Gavey enrolled 40 volunteers who were treated with HBO cycling continuously for 4 days in periods of 2-h exposures to 100% O_2_ at 2 ATA, followed by 1 h in room air at normobaric pressure (13). They concluded their report with the following statement “We think that the use of frequent, intermittent sessions of hyperbaric oxygen at 2 ATA, during the acute phase of the circulatory crisis shows promise of being a significant advance in treatment” (13).

We have previously reported that brief periods of hyperbaric oxygen cycling (OxCy; 100% O_2_; 2 ATA pressure; 90 min/day; 5 days/week for 4 weeks) enhanced the retention of transplanted mesenchymal stem cells (cardiomyoplasty) and improved cardiac function in a rat model of MI induced by ischemia-reperfusion (14). Comparisons were made to MI hearts receiving stem cell transplantation alone, and an additional MI group receiving OxCy alone. The data from these two comparative groups was intriguing, as both of these “control” procedures appeared to have near-equivalent benefit, albeit not as significant as combined therapy. Nevertheless, this prompted a number of questions, including: (i) Is 2 ATA pressure (hyperbaric) required for the beneficial effect? (ii) Is 100% oxygen concentration necessary or can lower concentrations be used? (iii) Is 60 or 90 min of oxygen cycling required, or can this also be reduced?

The following report documents our attempt to investigate a number of these issues, the goal of which was to determine the optimal conditions for OxCy treatment (environmental pressure, % oxygen, exposure time, etc.) that are required to prevent myocardial damage while also maximizing benefits and quality of life for the subject. We found out that, in general, oxygen cycling resulted in a significant reduction of infarct size and improvement of cardiac function. An optimal condition of 30-min OxCy of 95% oxygen + 5% CO_2_ under normobaric conditions was found to provide optimal cardioprotection.

## Materials and methods

### Materials

Primary and secondary antibodies for Akt and pAkt were purchased from Cell Signaling Technology (Danvers, MA) and GE Healthcare (Little Chalfont, Buckinghamshire, UK), respectively. Polyvinylidene fluoride (PVDF) membrane and molecular-weight markers were obtained from Bio-Rad (Hercules, CA). Antibodies specific for p53 and Bax, were purchased from Santa Cruz Biotechnology (Santa Cruz, CA). Enhanced chemiluminescence (ECL) reagents were obtained from Amersham Pharmacia Biotech (GE Healthcare-Piscataway, NJ). RIPA lysis buffer was obtained from Santa Cruz Biotechnologies. All other reagents, of analytical grade or higher, were purchased from Sigma-Aldrich, unless otherwise noted. Microcrystals of LiNc-BuO were synthesized as previously reported (15).

### Induction of myocardial infarction by ischemia-reperfusion (I-R) injury

Fisher F-344 rats (male, 150–200 g body weight, Charles River Laboratories) were used in this study. All procedures were performed with the approval of the Institutional Animal Care and Use Committee of The Ohio State University and conformed to the Guide for the Care and Use of Laboratory Animals, published by the National Institutes of Health (NIH Publication No. 86–23, Revised 1996). Animals were subjected to regional ischemia by ligation of the left-anterior-descending (LAD) artery for 60 min followed by reperfusion, as described previously (14). Briefly, an oblique 12-mm incision was made 2–3 mm dorsal to the sternum and run parallel to the 3rd and 4th intercostal space toward the vertebra. The chest cavity was opened using a retractor and the heart was visualized. Ischemia was induced by temporarily ligating the LAD artery for 60 min, followed by reperfusion by release of the ligature. Following I-R, the chest cavity was closed by bringing together the third and fourth ribs with 4-0 silk suture. The layers of muscle and skin were then closed with 4-0 polypropylene suture.

### Experimental groups

**Figure 1:**
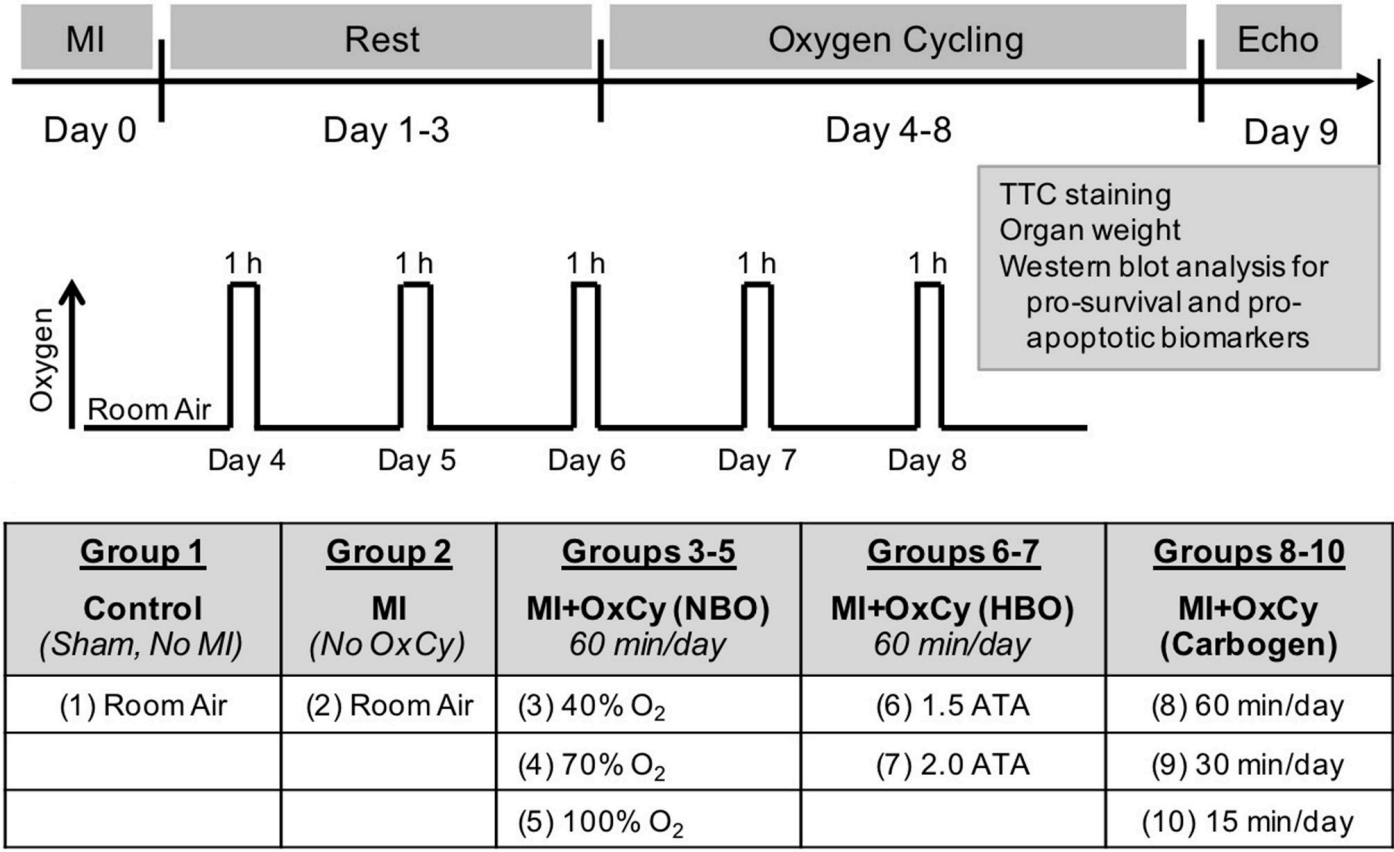
Experimental Setup. Myocardial infarction (MI) was induced by ischemia-reperfusion injury by temporarily ligating LAD artery for 60 min. OxCy treatment was initiated after 3 days of rest. The study was divided into 10 groups. **Group 1** (Control) was not subjected to MI or OxCy. **Group 2** (MI) was subjected to MI, but these animals were kept at room air without OxCy. **Groups 3-5** were subjected to 60 min of OxCy using 40, 70, or 100% O_2_ (balanced with N_2_), respectively, under normobaric conditions (NBO). **Groups 6-7** received 60 min of OxCy under hyperbaric oxygen (HBO) using 100% O_2_ at 1.5 or 2 ATA. **Groups 8–10** received OxCy using carbogen for 15, 30, or 60 min. Echocardiography and biomarker analysis were carried out after 5 days of OxCy treatment.

After allowing a period of 72 h for post-surgical recovery, experimental animals were treated using supplemental oxygen cycling (OxCy) by placing them in a custom-built chamber each day for 5 consecutive days under different treatment conditions. Acute myocardial infarction (MI) was induced by I-R injury as described above. Animals with MI were treated with 4 different concentrations of supplemental oxygen (40, 70, 90, and 100%) for a period of 60, 30, or 15 min per day, delivered under normobaric or hyperbaric pressure. The composition of oxygen and delivery durations were chosen to cover a broad range to represent the most effective to easily-applicable clinical conditions to arrive at an optimal treatment modality. The study was divided into a total of 10 groups with 5 major categories (Figure 1). Group 1: Control group that had neither MI nor subjected to oxygen cycling. Group 2: MI group had MI, but these animals were kept at room air all the time without OxCy. Groups 3–5 had MI treated with 60 min of OxCy at normobaric pressure and divided according to the concentration of supplemental oxygen; 40% O_2_, 70% O_2_, or 100% O_2_. Groups 6–7 had MI treated with 60 min of OxCy using 100% O_2_ in hyperbaric chamber and divided according to pressure 1.5, or 2 ATA. Groups 8-10 had MI treated with carbogen (95%O_2_ + 5% CO_2_) at normobaric pressure and divided based on the duration of treatment: 15, 30, or 60 min. Each group contained 4–12 rats.

### Cardiac function using echocardiography

Cardiac function was measured using M-mode echocardiography after 5 days of OxCy. Rats were anesthetized using 1.5–2% isoflurane in air, and M-mode ultrasound images were acquired using a Vevo 2100 high-resolution ultrasound imaging system (VisualSonics; Toronto, ON, Canada).

### Myocardial pO_2_ using *in vivo* EPR oximetry

After anesthetization followed by intubation, the chest of the animal was re-opened between the 3rd and 4th intercostal space, and the cavity was held open using a copper-based retractor wire. Using an in-house produced displacement syringe tip, a sample of LiNc-BuO EPR oximetry probe was implanted into the left ventricular myocardial tissue via a 25-guage needle. After ensuring that ventricular perforation did not occur, the rat was immediately transferred to a custom-built chamber (10-cm diameter and 40-cm length) with provisions for gas inlet and outlet. A surface-loop resonator was inserted through an air-tight port in the chamber and placed above the heart. An *in vivo* EPR oximetry unit (Magnettech; Berlin, Germany) was used for pO_2_ measurements. Baseline EPR measurements were made using room air (21% oxygen). When needed, hyperoxygen gas mixtures were passed through the chamber during measurements. EPR spectra were acquired as single 30-sec-duration scans. The instrument settings were: microwave frequency, 1.2 GHz (L-band), incident microwave power, 4 mW; modulation amplitude, 180 mG, modulation frequency 100 kHz; receiver time constant, 0.2 s. The peak-to-peak width of the EPR spectrum was used to calculate pO_2_ using a standard calibration curve (16).

### Myocardial infarct area using TTC staining

Heart tissues were frozen at −20°C for 10 min and cut into 4 slices transversely. One of the middle slices was then incubated at 37°C for 10 min with 1.5% TTC (triphenyltetrazolium chloride). Gross photographs were taken using a dissection microscope (Nikon). The images were analyzed by computerized planimetry using MetaVue image analysis software (Molecular Devices). The area of myocardial cross-section showing white color was defined as infarct, and the region in red was defined as the area at risk. Infarct size was expressed as a percentage of the area at risk.

### Protein molecular expression analysis using western blotting

Hearts were rapidly excised, rinsed in ice-cold PBS (pH 7.4), flash-frozen in liquid nitrogen, and stored at −80°C until analysis. Tissue homogenates were prepared from the anterior wall of the left ventricles of rats of each OxCy subgroup using a RIPA lysis buffer system. The proteins were separated by SDS-polyacrylamide gel electrophoresis, transferred to a PVDF membrane, and blocked with 5% nonfat milk powder (w/v) in TBST (10-mM Tris, 10-mM NaCl, 0.1% Tween 20) for 1 h at room temperature. The proteins were then probed for expression of Akt, and phospho-Akt (Ser-473) by incubating the membrane overnight at 4°C with the primary antibodies, followed by incubation with horseradish peroxidase (HRP)-conjugated secondary antibodies for 1 h. The membranes were then developed using an ECL system. The same membranes were then reprobed for glyceraldehyde 3-phosphate dehydrogenase (GAPDH). The protein intensities were quantified using an image-scanning densitometer (Scion Corporation, Frederick, MD), and standardized against the GAPDH signal. Protein expression levels were quantified using Image Gauge v. 3.45 software.

### Data analysis

The statistical significance of the results was evaluated using one-way ANOVA and Student's *t*-tests. The values were presented as Mean ± Standard Deviation (SD). A *p*-value of less than 0.05 was considered significant.

## Results

### Effect of hyperoxygenation on myocardial pO_2_

**Figure 2:**
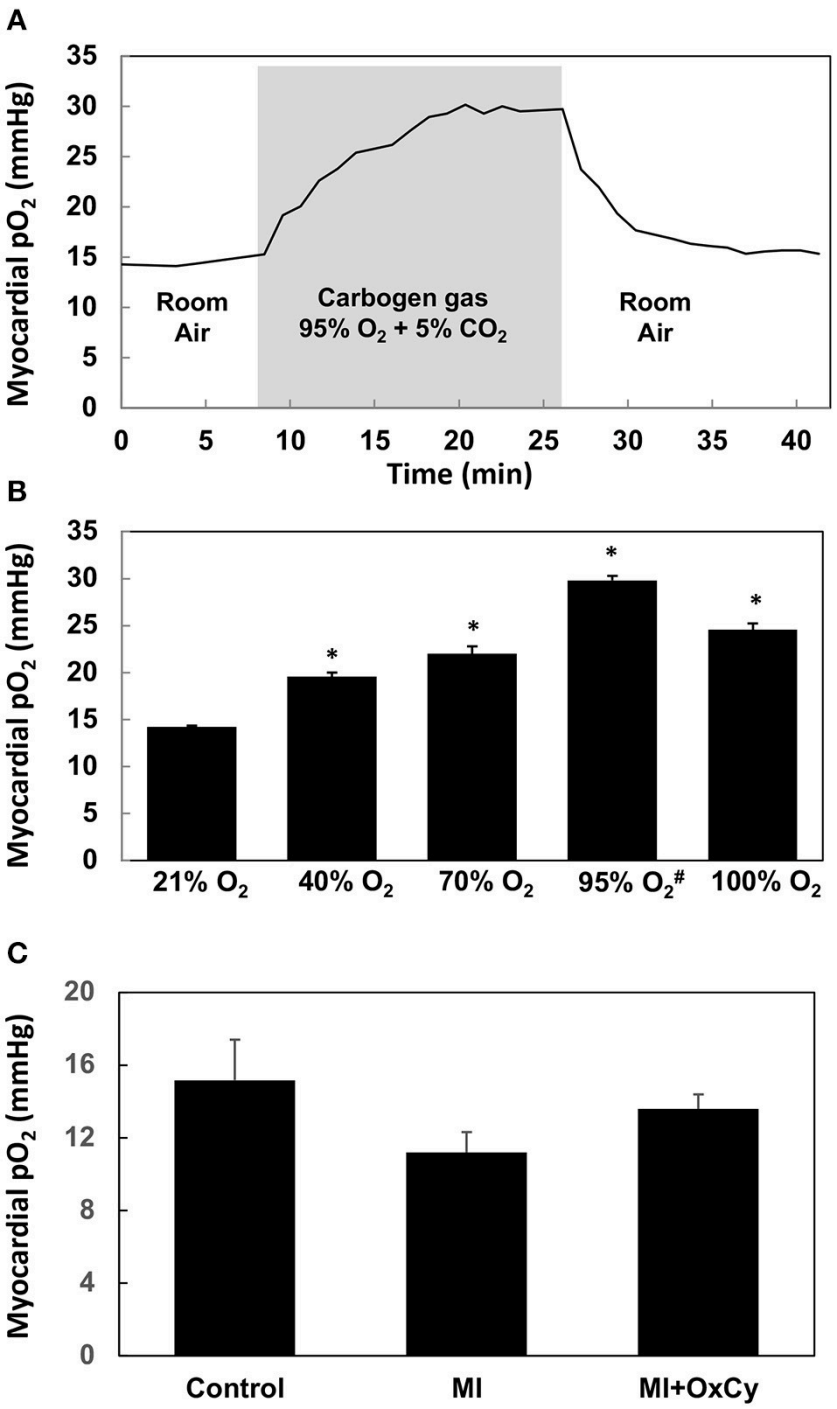
Myocardial pO_2_ in rat hearts obtained using *in vivo* EPR oximetry. LiNc-BuO oxygen-sensing crystals were implanted in the left ventricular myocardium. Myocardial pO_2_ was measured using EPR oximetry. Rats were intubated and allowed to breathe gas mixture containing the respective O_2_ levels along with 1.5% isoflurane anesthesia during EPR measurements. **(A)** Effect of carbogen breathing on myocardial oxygenation in healthy hearts. The myocardial pO_2_ increases from ~14 mmHg baseline level to about 28 mmHg after 18 min of carbogen administration and returned to baseline up on withdrawal of carbogen. **(B)** Peak myocardial oxygenation after about 20 min of administration of hyperoxygen gas mixed with nitrogen (^#^mixed with 5% CO_2_). Statistically significant increase in peak myocardial pO_2_ is observed in all hyperoxygen groups when compared to baseline (21% O_2_) group (*P < 0.01). Carbogen administration shows the largest increase. Data represent Mean ± SD (*N* = 3). **(C)** Myocardial pO_2_ in rat hearts subjected myocardial infarction (MI) and infarcted group treated with OxCy using carbogen for 60 min/day for 5 days (MI+OxCy), measured at 1-week post-MI. MI was not induced in the control hearts. Data represent Mean ± SD (*N* = 3). There were no statistically significant differences between the groups.

Using EPR oximetry, we determined the effect of increasing concentrations of inspired oxygen on myocardial pO_2_ in healthy (non-MI) rats. The myocardial pO_2_ increased from a baseline value of about 14 mmHg to levels peaking around 28 mmHg during exposure to carbogen (95% O_2_/5% CO_2_; Figure 2A). Myocardial pO_2_ reached its peak in about 12 min after start of carbogen administration and remained elevated above the baseline (room air) level after termination of carbogen and re-exposure to room air. We next determined the peak levels of myocardial pO_2_ observed during exposure of healthy (non-MI) rats to hyperoxygen ranging from 40–100% O_2_. The results showed a concentration-dependent increase in peak myocardial oxygenation, while carbogen exposure showed a significantly higher increase when compared to all other groups. Interestingly, exposure to 100% O_2_ showed a significant decrease in peak oxygenation when compared to carbogen (Figure 2B). Overall, the results indicated that exposure of rats to hyperoxygen increased myocardial pO_2_ during the period of administration. We further determined the effect of oxygen-cycling on myocardial pO_2_ in rats at 1-week post-MI. The results showed no significant differences in the myocardial pO_2_ values among the control, MI, and MI hearts treated with oxygen cycling (MI+OxCy; Figure 2C).

### Cardiac function, infarction, and remodeling in MI hearts treated with oxygen cycling

**Figure 3:**
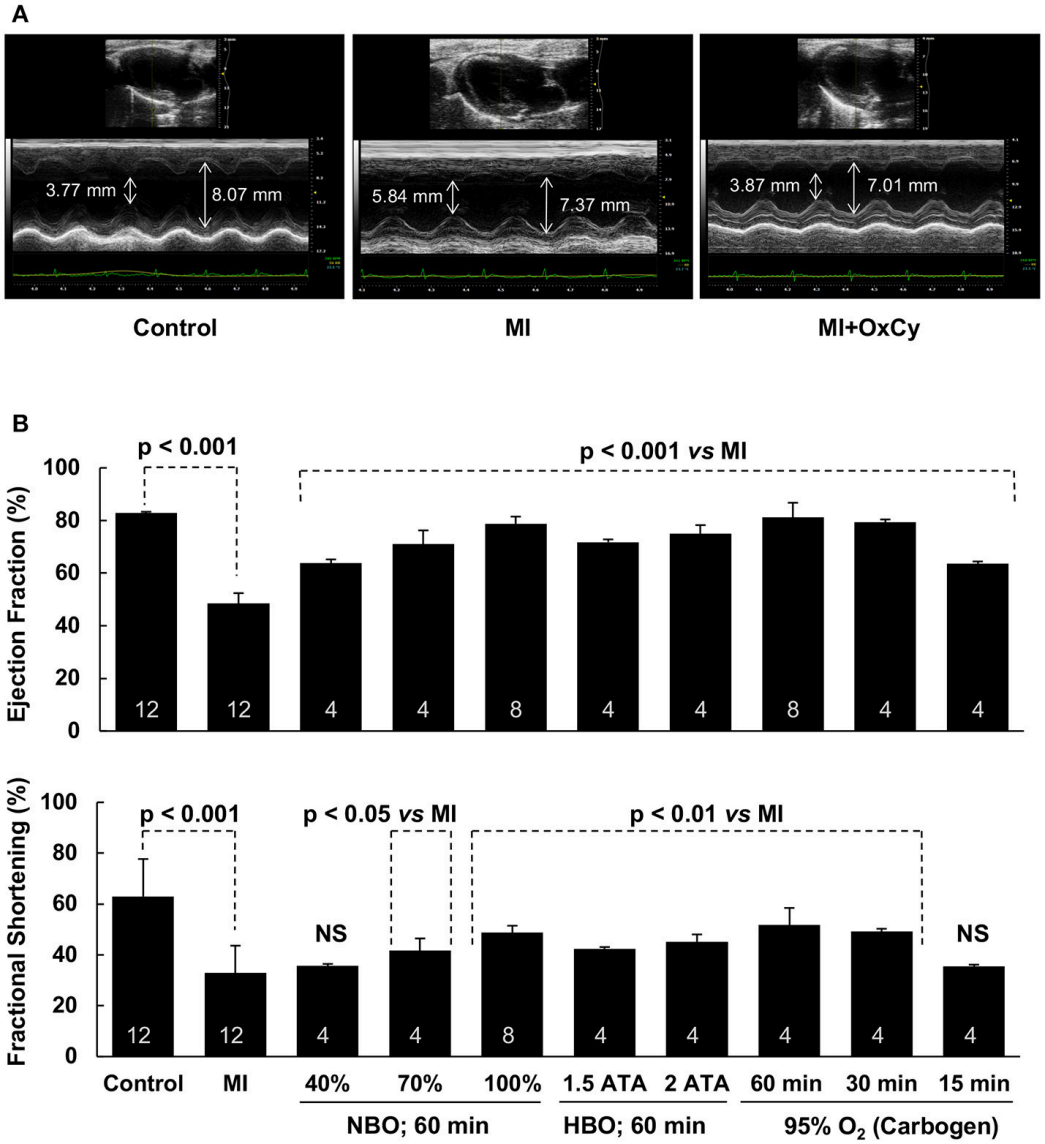
Recovery of cardiac function of MI hearts following OxCy. Cardiac function was measured using transthoracic M-mode echocardiography. **(A)** Representative echocardiogram of Control, MI, amd MI hearts treated with OxCy using 95% oxygen (carbogen) for 60 min/day for 5 days. Systolic and diastolic diameters are shown on the images. **(B)** Left ventricular ejection fraction and fractional shortening are shown for non-MI hearts (Control), untreated MI hearts (MI), MI hearts reated with OxCy for 1 h/day with 40% O_2_ (40%), 70% O_2_ (70%), 100% O_2_ (100%), 100% O_2_ at 1.5 ATA (1.5 ATA), 100% O_2_ at 2 ATA (2 ATA), 95% O_2_/5% CO_2_ (60 min), 30 min/day with 95% O_2_/5% CO_2_ (30 min), and 15 min/day with 95% O_2_/5% CO_2_ (15 min). Data represent Mean ± SD. Number of hearts per group is shown on the bar. Statistical significance, as indicated; NS indicates not significant when compared to MI group.

To determine the effect of oxygen cycling on cardiac function, we used M-mode echocardiography on day 9 after induction of MI. Significant decreases in ejection fraction and fractional shortening were observed in the infarct (MI) hearts that did not receive oxygen-cycling treatment (Figure 3). Rats treated with all levels of hyperoxygenation, both at normobaric and hyperbaric pressures, showed variable levels of recovery of cardiac function depending on the inspired oxygen level. Rats subjected to hyperoxygenation (100% O_2_) at 1.5 or 2 ATA pressure, 1 h/day for 5 days, demonstrated notable recovery of cardiac function; however, improvement at these hyperbaric levels was not significantly better than that at ambient pressure. In fact, their respective ejection fraction and fractional shortening means were less than those at 1 ATA. Interestingly, rats subjected to either normobaric 100% O_2_ or 95% O_2_/5% CO_2_ (carbogen) for the same time duration showed comparable levels of cardiac functional recovery after MI. Recovery of MI hearts treated with 1 h of carbogen were not significantly different when compared to hearts treated with 1 h of 100% oxygen. The carbogen treatment further revealed that 30 min/day administration for 5 days did not show any significant difference in the cardiac function when compared to 1 h/day carbogen administration. However, 15 min/day of carbogen treatment did not yield similar functional improvement and it resulted in significantly less ejection fraction and fractional shortening when compared to 30 min/day treatment.

**Figure 4:**
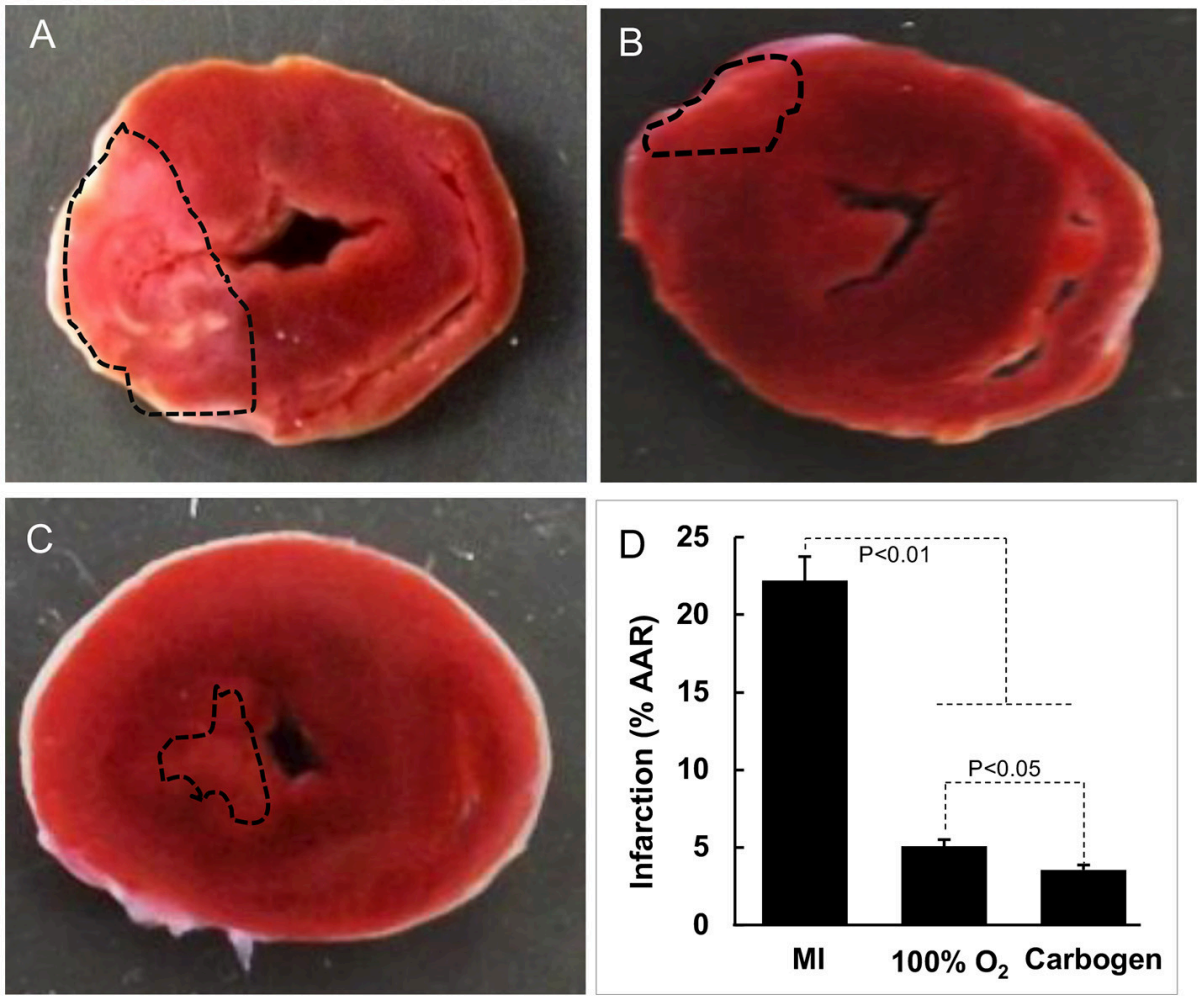
Effect of oxygen-cycling on myocardial infarction. Representative images of triphenyltetrazolium chloride (TTC)-stained sections of rat heart are shown. **(A)** MI heart without treatment. **(B)** MI heart treated with 100% O_2_ for 60 min/day for 5 days). **(C)** MI heart treated with carbogen (100% O_2_ + 5% CO_2_) for 30 min/day for 5 days. **(D)** Infarct size expressed as % of area at risk (AAR). Data represent Mean ± SD (*N* = 3).

**Figure 5:**
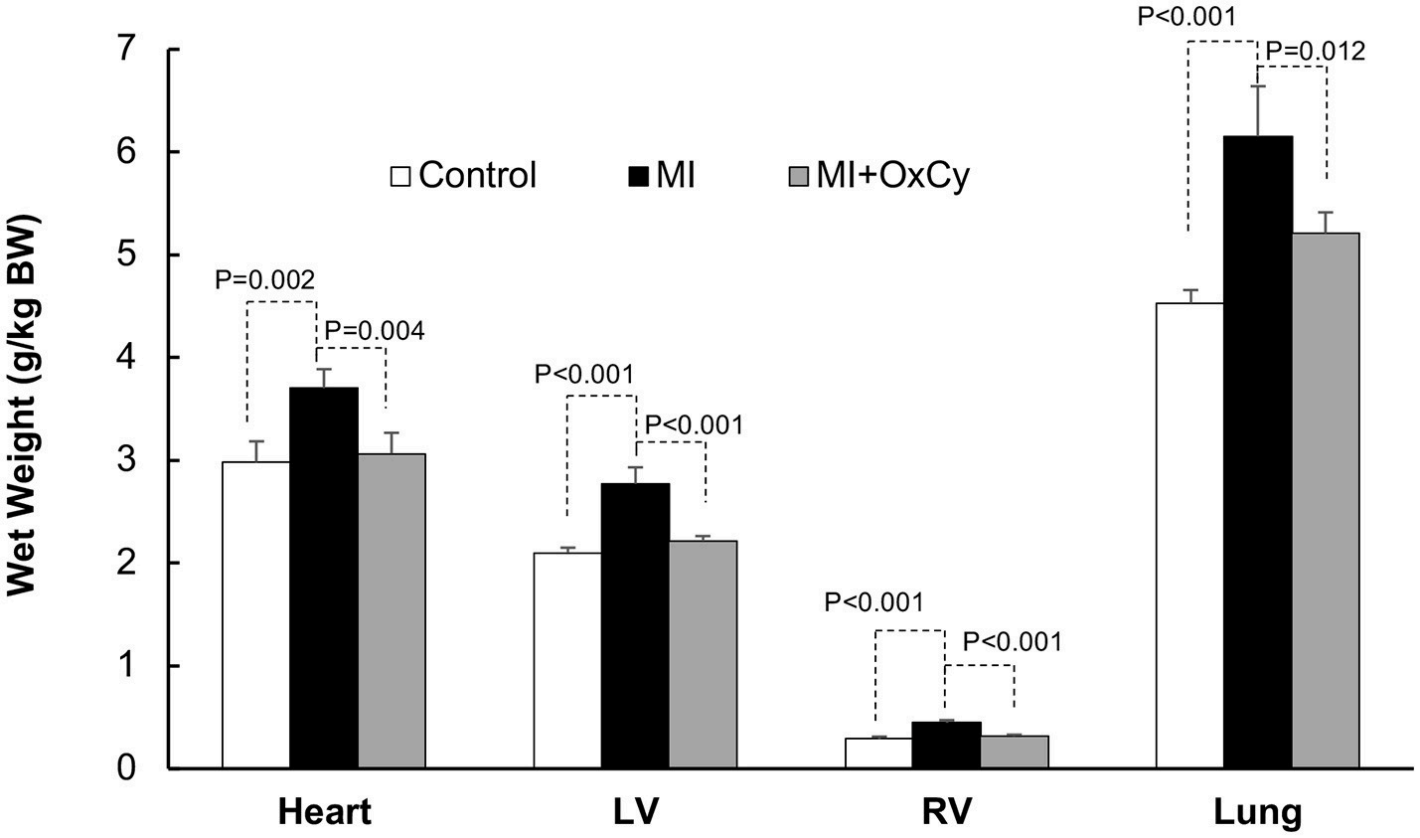
Effect of oxygen-cycling on cardiac remodeling in the MI heart. Cardiac remodeling was assessed using wet weights of the whole heart, whole lung, right ventricle, and left ventricle with septum. The results showed a significant increase in the weight of the organs in the MI group which was inhibited by oxygen-cycling treatment with 95% O_2_/5% CO_2_ for 30 min/day.

Postmortem analysis of cardiac tissue with TTC staining was consistent with the echocardiography data, with substantial reduction of infarct area in MI hearts after oxygen cycling with 95% O_2_/5% CO_2_, 30 min/day (Figure 4). Acute MI, induced by surgical I-R, is known to cause myofibrillar edema leading to myocardial dysfunction (17). To evaluate the effect of supplemental oxygenation on cardiac edema, we measured wet weights of whole heart, whole lung, right ventricle and left ventricle with septum. The results showed a significant increase in the weight of the organs which was inhibited by oxygen-cycling treatment with 95% O_2_/5% CO_2_ for 30 min/day (Figure 5).

### Expression of key signaling proteins in MI hearts subjected to oxygen cycling

**Figure 6:**
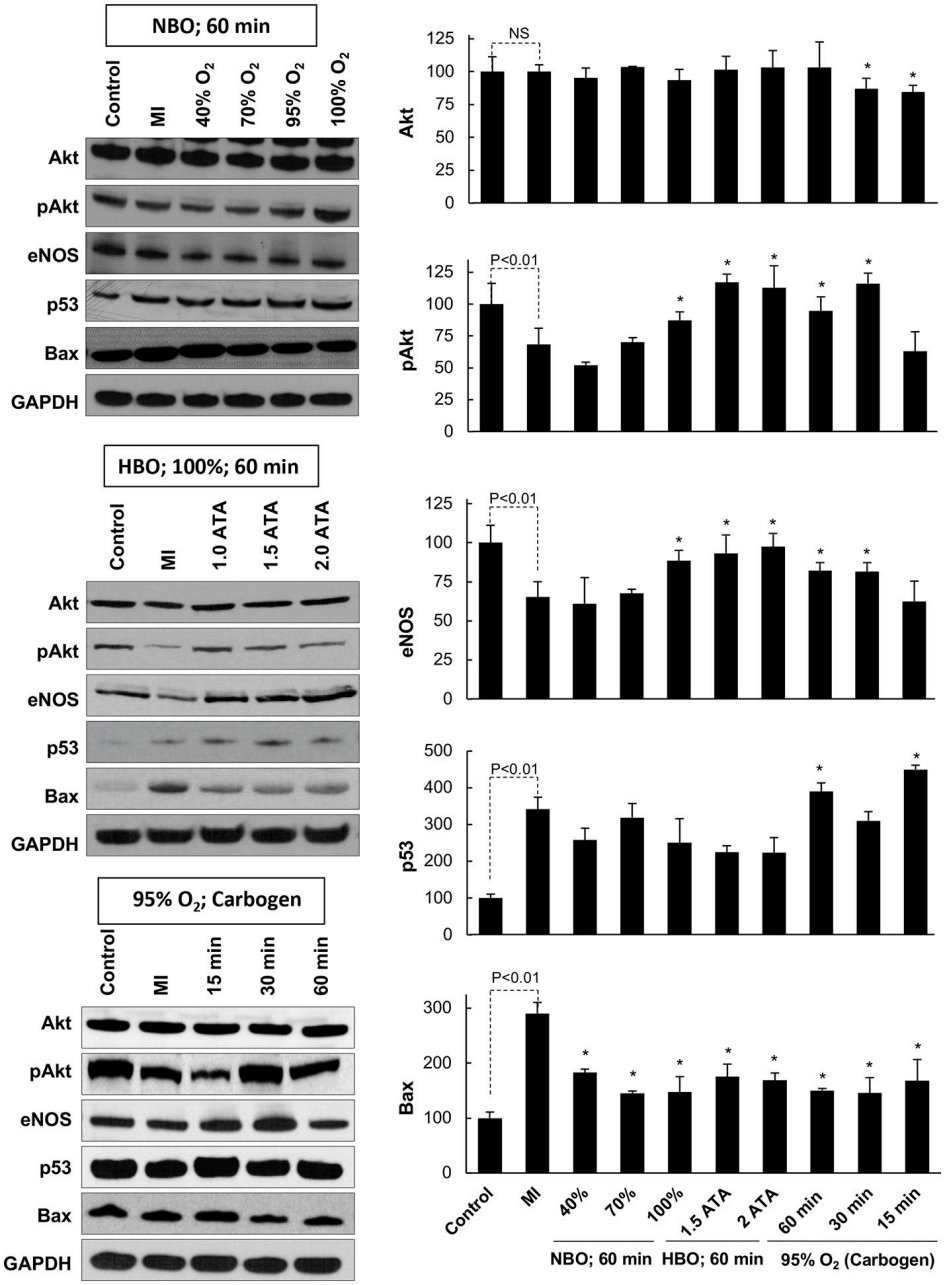
Key signaling proteins in the MI heart subjected to OxCy. The levels of Akt, pAkt, eNOS, p53, and Bax were probed in hearts subjected to daily oxygen cycling 3 days after induction of MI for 5 days. Representative western blot images are shown on the left and densitometric analyses (band intensity) of Akt, pAkt, eNOS, p53, and Bax are shown on the right. Results are plotted as Mean ± SD for each group, expressed as a percent of the respective Control group. The number of hearts (N) used for densitometric analysis: Control, 5–7; MI, 11–15; all other groups, 3–10. Statistical significance between Control and MI group is indicated (NS denotes not significant). *p < 0.05 vs. MI group.

To understand the underlying signaling pathways involved in the cardiac recovery after oxygen cycling, we analyzed heart tissues using Western-blot assay. We analyzed the following pro-survival and pro-apoptotic proteins, namely, Akt, eNOS (NOS3), pAkt, p53, and Bax, which we have shown to be critically involved in MI hearts subjected to OxCy (18). The experimental groups included non-infarcted healthy hearts, infarcted hearts, and each of the different treatments (Figure 6). The blot images were quantified, and data normalized to each group's respective control group (Figure 6). Akt levels remained unchanged across all groups. MI hearts showed a significant reduction in the pro-survival protein pAkt when compared to control hearts. Treatment with 100% oxygen at 1-2 ATA as well as with 30 and 60 min of carbogen significantly increased pAkt expression. In comparison to MI hearts, we found significantly increased levels of the pro-survival protein eNOS in the hearts treated with 100% oxygen at 1, 1.5, and 2 ATA pressures as well as in hearts treated with carbogen for 30 or 60 min. There did not appear to be any trend in p53 levels across the different treatment groups; however, it is relevant to note that the infarcted hearts as well as nearly all treated hearts displayed overall higher expression of p53 following oxygen cycling than the control hearts. The pro-apoptotic protein Bax was significantly amplified in MI hearts when compared to control hearts, while all of the treated groups showed significantly reduced Bax levels.

## Discussion

The effectiveness of hyperoxygenation treatment post-MI, whether under normobaric or hyperbaric conditions, has been a highly debated topic, despite widespread use over the past century (19, 20). Two recently-published reviews found a very limited number of randomized, controlled trials of supplemental oxygen vs. normal air for patients with MI. In both reviews, the authors concluded that the studies showed no benefit of inhaled oxygen therapy (11, 21, 22). These reviews were cited in a recent editorial by Dr. C. Richard Conti, who states, “After reviewing the literature, I was unable to find hard evidence that the use of supplemental oxygen (hyperbaric or normobaric) in an uncomplicated acute myocardial infarction (AMI) is beneficial, and there is some evidence that it may be harmful” (23). Factors to consider when reviewing prior work are the timing and sequence of hyperoxygenation/HBO treatment and concentration of oxygen delivered. In both the 1969 study by Ashfield and Gavey and the 1976 study by Rawles, patients were treated within 24 h of MI presentation (13, 24). In another clinical study, patients were treated with normobaric hyperoxygenation following thrombolysis within 6 h following MI (25). The patients receiving oxygen treatment were given 100% oxygen delivered via face mask for a period of 24 h (25). After mixing with room air, it is approximated that face mask delivery of 100% oxygen produces an FiO_2_ (fraction of inspired oxygen) of 40%.

In a study using rats, dos Santos et al. (26), subjected experimental animals to a single, 1-h period of HBO (100% O_2_ at 2.5 ATA) immediately following coronary occlusion and observed a decrease in the necrotic area and acute mortality. In the more recent “HOT MI” clinical study (12), patients with acute MI underwent a single application of HBO immediately following thrombolysis. The results showed a 10% increase in ejection fraction in the HBO group, rapid pain resolution and attenuated creatine phosphokinase, although the results were not statistically significant. Gilmour et al. (27), subjected dogs to a brief period (20 min) of 3 ATA HBO prior to occlusion of the coronary artery followed by a second period of HBO immediately after occlusion and concluded that HBO did not improve left ventricular function impaired by MI. The results of this study are difficult to interpret, as it has been reported that tissue oxygen tensions remain elevated after HBO treatment has concluded. Increased oxygen tension following the first HBO period would likely have increased the levels of reactive oxygen species formed during the ischemic/occlusive period, resulting in more damage to the cardiac tissues than would have occurred under normal circumstances in which normal air breathing occurs prior to MI. Cabigas et al. (28) have reported that HBO treatment prior to the induction of IR injury limited myocardial damage and decreased infarct size, which was attributed to increased levels of nitric oxide.

In the current study and in our prior work (14), hyperoxygenation or HBO treatment was started 3 days post-MI in experimental rats. This recovery period was introduced to allow healing for the pneumothorax created by the MI-induction procedure. Tension pneumothorax is a contraindication for clinical HBO therapy (29). Non-HBO animals were also allowed this recovery period to avoid complicating the study by having different treatment starting points. Our group has also reported that re-establishment of coronary perfusion following simulated MI in a rat model produces a hyperoxygenation in the affected region of myocardial tissue for up to 24 h, and possibly longer (30). This recovery period would allow the damaged myocardial tissue to return to a steady-state perfusion condition.

Western-blot studies showed that hearts subjected hyperoxygen cycling, particularly carbogen, showed significantly higher expression of the pro-survival proteins pAkt and eNOS and a lower expression of the pro-apoptotic protein Bax when compared to MI hearts. Interestingly, the p53 level was significantly higher in the carbogen groups (60 and 30 min) when compared to MI group. These results are consistent with our previous reports that used a similar experimental model with hyperbaric oxygen cycling (14, 18). In the study by Gogna et al. (18), we found a dual role for p53, which, depending on oxygenation, can elicit apoptotic death signals or NOS3-mediated survival signals in the infarct heart. It was observed that p53 exhibited a differential DNA-binding, namely, BAX-p53RE in the infarct heart or NOS3-p53RE in the oxygenated heart, which was regulated by oxygen-induced, post-translational modification of p53. In the infarct heart, p53 was heavily acetylated at Lys-118)- residue, which was exclusively reversed in the oxygenated heart, apparently regulated by oxygen-dependent expression of TIP60. The inhibition of Lys(118) acetylation promoted the generation of NOS3-promoting prosurvival form of p53. Thus, oxygenation was found to switch p53-DNA interaction by regulating p53 core-domain acetylation, thereby promoting a prosurvival transcription activity of p53. We assume a similar mechanism may be operative in the current study.

Many prior animal or clinical studies applied hyperoxygenation or HBO as a one-time intervention (12, 31–34), or for 24 h or more continuously (24, 35). When compared to other hyperoxygenation or HBO protocols, the “oxygen cycling” used in the current study is unique. The only prior work which used a periodic or cyclic approach was the Ashfield and Gavey study, in which treated patients were subjected to 2 h of HBO in 100% oxygen, followed by 1 h in air at normobaric pressure (13). This cycle was repeated continuously for an average period of 4 days. As noted by Jain, “Repeated exposure to HBO at intervals insufficient to allow total recovery from pulmonary oxygen toxicity may lead to cumulative effects” (18). Heeding this warning, the cyclic process employed in the current study, in which a single 1-h dive was administered daily, allowed sufficient time for recovery between subsequent applications of hyperoxygenation.

In the present study, HBO administered at 1.5 or 2 ATA did not provide significantly better results with regard to recovery of cardiac function when compared to 100% oxygen treatment administered at ambient pressure. For this reason, we conclude that hyperbaric conditions, while beneficial overall, is not entirely necessary or even practical for oxygen therapy, especially considering a patient in a clinical setting would need to be placed inside a chamber to synthesize such an environment. Carbogen, a mixture of 95% O_2_ and 5% CO_2_, was found to enhance myocardial tissue oxygenation to the greatest extent, and this increase was notably greater than when 100% oxygen was used. This is most likely due to two well-documented hemodynamic responses: (i) hyperoxygenation promotes vasoconstriction and (ii) CO_2_ is a potent vasodilator. From these results, we contend that the vasodilatory effect of CO_2_ outweighs the vasoconstrictive effect of hyperoxygenation under carbogen breathing. If the foremost objective of supplemental oxygen therapy as an adjuvant measure for MI patients is to increase myocardial tissue oxygenation, then the use of carbogen, instead of 100% oxygen, should be considered.

Testing various treatment conditions led to the conclusion that carbogen breathing at ambient pressure is arguably the ideal scenario for treating MI. Administration of carbogen for 1 h led to improved ejection fraction and fractional shortening. Furthermore, it is important to note that the 30- and 60-min carbogen-breathing groups did not show any significant differences in cardiac function, which suggests that a half-hour of carbogen treatment is sufficient to produce substantial improvements. In conclusion, the oxygen-cycling therapy serves as a very appealing option for myocardial infarction treatment because it yields some of the greatest benefits while also minimizing treatment time and inconvenience for the subject.

## Author contributions

PK: Design, interpretation and writing; AP: Writing; MLK: Experiments—protein analysis; SN: Experiment—Surgery, MI, echo; SM: Experiment—Surgery, MI, echo; PR: pO_2_ measurements; AD: Data analysis; MK: Experiment desin, interpretation; BR: Experiment design, pO_2_ studies.

### Conflict of interest statement

The authors declare that the research was conducted in the absence of any commercial or financial relationships that could be construed as a potential conflict of interest. The handling Editor declared a shared affiliation, though no other collaboration, with several of the authors SN, SM, PR, AD, MK, BR, at the time of the review.
